# Editorial: Novel Drugs Targeting the Microenvironment and the Epigenetic Changes in Hematopoietic Malignancies

**DOI:** 10.3389/fphar.2020.614614

**Published:** 2020-12-10

**Authors:** Diana Gulei, Ciprian Tomuleasa, Liren Qian, Cristina Bagacean, Carlo M. Croce, Gabriel Ghiaur

**Affiliations:** ^1^ Medfuture Research Center for Advanced Medicine, Iuliu Hatieganu University of Medicine and Pharmacy, Cluj Napoca, Romania; ^2^ Department of Hematology, Ion Chiricuta Clinical Cancer Center, Cluj Napoca, Romania; ^3^ Department of Hematology, Medfuture Research Center for Advanced Medicine, Iuliu Hatieganu University of Medicine and Pharmacy, Cluj Napoca, Romania; ^4^ Department of Hematology, The Sixth Medical Center, Chinese PLA General Hospital, Beijing, China; ^5^ INSERM UMR1227, B lymphocytes and autoimmunity, University of Brest, Brest, France; ^6^ Service d’Hématologie, Centre Hospitalier Régional et Universitaire de Brest, Brest, France; ^7^ Department of Molecular Virology, Immunology and Medical Genetics, Ohio State University, Columbus, OH, United States; ^8^ Department of Leukemia, Sidney Kimmel Comprehensive Cancer Center, Johns Hopkins University, Baltimore, MD, United States

**Keywords:** tumor microenvionment, epigenetic changes, hematological malignances, methylation, pharmacology

Starting with the first definition of epigenetics “to understand how the genotypes of evolving organisms can respond to the environment in a more co‐ordinated fashion” originated in the research of Conrad Hal Waddington (1942) until current days, the field of epigenetics has evolved greatly (Dimopoulos and Gronbaek, 2019). We are now aware not only of the substantial contribution of the epigenomic patterns in development and homeostasis, but also of the profound implications of epigenetic alteration in disease pathogenesis (Stahl et al., 2016). Hematological malignancies light the way and provided proof-of-concept not only for the unraveling the role of epigenetic alterations in disease, but also for correcting these alterations and the clinical development of novel therapies (DNA hypomethylating agents and histone deacetylase inhibitors) for patients with myelodysplastic syndromes (MDS) and acute myeloid leukemias (AML). Large scale studies of cancer cells show an epigenetic drift toward global hypomethylation with enhances chromosomal instability or intensified hypermethylation at CpG islands within the promoter of tumor suppressor genes and sustained tumorigenesis (Eden et al., 2003; Herman and Baylin, 2003; Karpf and Matsui, 2005; Esteller, 2008). On top of this, further research has shown that epigenetic mechanisms have deep implications for the establishment of cancer permissive microenvironments (Maio et al., 2015). The deploy of epigenetic alterations has been shown to hijack the mechanisms of immune surveillance in order to escape the antitumor immune responses (Cao and Yan, 2020). In this sense, by remodeling the tumor microenvironment, a combination between immunotherapy and epigenetic agents may provide clinical benefit for patients with incomplete responses to immunomodulatory agents (Villanueva et al., 2020). In addition, efforts are undertaken to understand the role of epigenetic alterations and tumor microenvironment in reshaping the metabolic fitness and chemoresistance of cancer cells (Carrer and Wellen, 2015; Forte et al., 2019). There is increasing evidence that the tumor microenvironment is essential for maintaining malignant hematopoiesis. Since stromal elements (i.e., cancer-associated fibroblast, endothelial cells and other) do not exhibit somatic mutations, they are likely to be corrupted by the malignant cells via epigenetic driven events (Sylvestre et al., 2020). To this point, mesenchymal stromal cells from AML show focal points of DNA hypermethylation, but also global hypomethylation compared to their normal counterparts (von der Heide et al., 2017). Therefore, epigenetic therapy could benefit patients with hematological malignancies not only due to reprograming the cancer cells, but also by rescuing the tumor microenvironment.

The Research Topic entitled *Novel Drugs Targeting the Microenvironment and the Epigenetic Changes in Hematopoietic Malignancies* covers recent advancements in our understanding of the role of the microenvironment in hematological malignancies. To this end, response to epigenetic drugs are presented within a cohort of juvenile myelomonocytic leukemia (JMML) cases that were molecularly annotated via targeted next generation sequencing (NSG). Treatment with 5-Azacitydine was well tolerated and had effective results in both *de novo* JMML and relapsed patients (Marcu et al., 2020). The authors are highlighting the paramount role of microenvironment not only in disease progression and treatment response, but also in the outcomes of bone marrow transplantation in these patients. These data emphasize the importance of establishing a healthy substrate for normal hematopoiesis in patients with MDS undergoing bone marrow transplantation. The article is expending on the alterations that intervene in cancer associated stromal cells and how different therapies, including epigenetic agents can modulate the malignant environment (Teodorescu et al., 2020). The heterogeneity of the tumor microenvironment within AML is discussed with a focus on how unique immune profiles can serve as a surrogate distinct prognosis profiles for patients with hematological malignancies. Whether or not these changes are related to genetic and epigenetic events remains to be discussed (Antohe et al., 2020). The concept of minimal residual disease (MRD) in oncological hematology is presented within this research topic (Radu et al., 2020). In addition, DNA methylation patterns detected via revolutionary surface-enhanced Raman spectroscopy (SERS) are used as quantifiable biomarkers of circulating tumor cells in liquid biopsies (Turcas et al., 2020).

Although the critical influence of the epigenetic landscape on cancer cells survival and development has been recognized, so is its role in the establishment of supportive tumor environments. Nevertheless, the heterogeneous clinical response of hematological malignancies patients to epigenetic therapy suggests a complex relation between epigenetics and cellular behavior. Perhaps the newly emerging field of epitranscriptomis may provide the missing link between modulation of gene expression and the malignant phenotype. To this end, N^6^-methyladenosine (m^6^A) is the most common non-genetic alteration in mRNAs. M^6^A impacts RNA metabolism and thus, mediates aberrant gene expression seen in disease development (Chen et al., 2019). RNA methylation is mediated by m^6^A methyltransferases, removed by demethylases and identified by m6A binding proteins, all of them recognized as “writers,” “erasers,” and “readers” respectively (Lan et al., 2019). The mechanism of RNA methylation in solid and hematological tumors is in its early years, without comprehensive clarification, and whatever this mechanism can surpass epigenetic changes is unknown. m^6^A enhances the translation of PTEN, BCL2, and c-MYC in AML (Vu et al., 2017), where YTHDF2 (m^6^A binding proteins) increases the expression of Tal1 (Li et al., 2018). On top, FMR1 (protein from the m^6^A binding complex) can bind multiple mRNAs to impair their translation and function (Edupuganti et al., 2017). However, how the entire RNA methylation machinery is functioning in cancer is still unknown; available data are showing distinct expression profiles of various “writers,” “erasers” and “readers” between cancer patients and distinct global m^6^A methylation profiles (Chen et al., 2019). The heterogeneity of these molecular profiles is demonstrating that RNA methylation is a dynamic process that can shift toward malignant favoring mechanisms, including during epigenetic treatment. Therefore, hematological patients that are non-responders to demethylating agents could be characterized by a dominant RNA methylation profile that favors the cancer phenotype or could present compensatory feedback mechanisms at the level of RNA to impede the modifications from the DNA induced by epigenetic therapy. Wide screening of different markers from the RNA methylation machinery could predict the eligible patients for epigenetic treatment, while analysis of the molecular background of the non-responder patients could offer new insights into the mechanisms of dynamic RNA methylation in cancer.

## Author Contributions

All authors listed have made a substantial, direct, and intellectual contribution to the work and approved it for publication.

## Funding

DG and CT are supported by 3 grants from the Romanian Ministry of Research and Innovation: Postdoctoral Research Projects 2020–2022 ‐ grant number PN-III-P1-1.1-PD-2019-0805, contract number PD 122/2020); CCCDI-UEFISCDI, Project No. PN-III-P4-ID-PCCF-2016-0112 within PNCDI III, as well as by awarded for Young Research Teams 2020-2022 (Grant No. PN-III-P1-1.1-TE-2019-0271), as well as by an international collaborative grant of the European Economic Space between Romania and Iceland 2020-2022 (Grant No. 19-COP-0031). GG is funded by K08 HL127269 (GG), R03 HL145226 (GG), P01CA225618 (GG), and P30 CA006973. LQ is funded by a grant from the National Natural Science Foundation of China (Grant No. 81800180) and the National Young Elite Scientists Sponsorship Program by the China Association for Science and Technology (Grant No. 17-JCJQ-QT-032).

## Conflict of Interest

The authors declare that the research was conducted in the absence of any commercial or financial relationships that could be construed as a potential conflict of interest.
